# What if 0 is not equal to 0? Inter-personal health utilities anchoring using the largest health gains

**DOI:** 10.1007/s10198-022-01537-8

**Published:** 2022-11-08

**Authors:** Michał Jakubczyk

**Affiliations:** SGH Warsaw School of Economics, Decision Analysis and Support Unit, Warsaw, Poland

**Keywords:** Health state utility values, Quality-adjusted life years, Interpersonal utility comparisons, Time trade-off, Cost-utility analysis, C44, D71, I18

## Abstract

Prioritizing health technologies requires comparisons of improvements in longevity or quality of life (QoL), or both. For this purpose, value sets are constructed that contain weights assigned to health states based on societal preferences. I show that how this is typically done may distort the results by giving unjustifiably larger impact to individuals who prioritize improvements in QoL over longevity. The problem results from equating the utility differences between being dead and full health across people, ignoring the fact that interpersonal utility comparisons are forbidden (or at least problematic) in economics. I propose another approach: the numerical value of maximal health gain (either in longevity or QoL) is assumed to be equal across individuals, to remove the impact of the range of utilities differing between people. I test this approach using EQ-5D-5L and EQ-5D-3L utilities elicited in Poland for two modeling techniques: a simple econometric model and a Bayesian one that accounts for censoring. The proposed approach increases the utilities of the worst health state: from $-0.41$ to $-0.25$ in EQ-5D-5L and from about $-0.54$ to $-0.36$ in EQ-5D-3L. In the Bayesian approach, the impact is greater: from $-0.45$ to $-0.11$ for EQ-5D-5L and from $-0.54$ to $-0.22$ for EQ-5D-3L. I discuss some normative arguments why the proposed approach may be more justifiable for aggregating individual preferences for health outcomes.

## Introduction

Averaging numbers using an arithmetic mean, simple as it is, must sometimes be used with caution. The examples range from school tasks about a car travelling both ways with different speeds to the Simpson paradox when averaging proportions in individual arms of clinical trials being pooled [1]. Another simple and deliberately somewhat grotesque example, illustrative of the problem being solved in this paper, is the dilemma of a family with two children, who want to allocate 18 days of holidays between the seaside and the mountains. A daughter and a son would prefer, respectively, a $12+6$ and a $6+12$ split. The parents conclude that since the kids would like to spend 50% and 200%, respectively, as much time in the mountains as at the seaside, it is fair to use the average of 125%. Hence, an 8+10 split is recommended. Obviously, this approach and result seem clearly unfair (and it would not likely occur in reality), because they break the *a priori* symmetry between the preferences of both children. The counter-intuitive outcome hinges on an arbitrary selection of a reference point to calculate the relative values to be averaged, i.e. the preferred amount of time to be spent in the seaside. As I show in the paper, an equivalent problem is lurking in health state utility elicitation.

The prioritization of health technologies to be reimbursed from the public budget is often based on cost-utility analyses (CUAs) [2, 3]. CUAs require the measuring of health outcomes, captured as changes in the length of life or health-related quality of life (HRQoL), or both. To operationalize the measurement of HRQoL, a descriptive system to define health states is introduced, often one of the EQ-5D family of instruments (introduced further below) [4]. The states are assigned numerical values, expressing how good or bad they are as perceived by society. The collection of values is referred to as *value set*. Then, the benefits of using the health technologies can be compared across diseases and types of outcomes.

To construct a value set, various empirical tasks are employed to infer how individuals perceive health states. A common approach, which I focus on in this paper, is the time trade-off method (TTO) [5]. In TTO, a respondent is asked to compare living in state *Q* for 10 years, denoted as (*Q*, 10), with living in full health (FH) for *T* years, denoted as $(\textit{FH},T)$. The desired result is the value of *T* that leads to indifference between the two alternatives. Typically, a quality-adjusted life years (QALY) model is assumed, in which the preferences over health profiles (a standard notation is used: $\prec$, $\preceq$) can be represented with von Neumann-Morgenstern (vNM, [6]) utilities. Additional assumptions are used for which the utility of (*Q*, *T*) can be decomposed as $u(Q)\times T$ [7, 8]. A function $u(\cdot )$, representing an individual’s preferences, is scaled so that $u(\textit{FH})=1$ and $u(\textit{dead})=0$ (henceforth, referred to as QALY scale). Then, based on TTO results, we estimate $u(Q)=T/10$. In the present paper, it is often more convenient to consider disutility of *Q* relative to FH, denoted by $\delta (Q)=1-u(Q)$.

For some *Q*, a respondent may consider $(Q,10)\prec (\textit{FH},T)$ for any $T\ge 0$. Such *Q* is denoted as worse than dead (WTD), and a modified TTO is needed to elicit *u*(*Q*). Historically, respondents were asked to compare immediate death with a mixed profile lasting for 10 years: $(\textit{FH},T)$ followed by $(Q,10-T)$ [9]. Because of some problems with this approach (elaborated further, also see [10]), a lead-time TTO method (LT-TTO) was introduced. In LT-TTO, 10 years in FH are added to both alternatives in the regular TTO to enable further trading: the respondents compare $(\textit{FH},T)$ with $(\textit{FH},10)$ followed by (*Q*, 10), $0\le T\le 10$. When indifference is reached, $u(Q)=\nicefrac {(T-10)}{10}$. No further trading beyond $T=0$ is possible, which leads to censoring of utilities at $-1$. The combination of regular TTO and LT-TTO is referred to as composite TTO (cTTO) [11]. It is commonly used to construct value sets for EQ-5D family of instruments [12–14] in a standardized protocol [15].

The utilities elicited for individual respondents are used to construct a value set: in simple terms by averaging the values for a single state between the respondents (and in more technical terms, by using econometric modeling, described further in the paper). In this paper, I show how this standard approach effectively places more impact on the final values to those individuals who value HRQoL so much they find some states worse than dead. In effect, this approach biases the elicited utilities downwards, or—putting it differently—it biases upwards the importance of HRQoL improvements. The bias results from what I claim is an arbitrary selection of the reference value when averaging utilities between the respondents. I propose an alternative approach, which makes the impact of individuals on the final value set more equal. I illustrate the impact of the proposed approach using two datasets with health state utilities elicited in Poland [13, 16] for EQ-5D-5L and EQ-5D-3L. I present some normative arguments why the proposed approach may be more justifiable as the basis of public decisions making.

The illustration is primarily focused on the EQ-5D-5L descriptive system. The health states are defined with five dimensions: mobility (MO), self-care (SC), usual activities (UA), pain/discomfort (PD), and anxiety/depression (AD). Each dimension can be at one of five levels, representing various situations from no problems to severe problems [17]. The levels are denoted with digits (1, ..., 5) and a five-tuple of digits (typically without parentheses or commas), e.g. 12321, denotes a health state. Hence, 11111 can be understood to represent FH, and the worst possible state (55555 for EQ-5D-5L) is called a *pits state*. In EQ-5D-3L, there are three levels per dimension, and 33333 is the pits state.

In Sect. "The problem and an idea of a solution", I present a simple illustrative example of the problem and solution in non-technical terms. In Sect. "Formal model specification", I present a formal model specification allowing to apply the solution to actual datasets. I use several model specifications to account for various sets of assumptions and demonstrate the robustness of the findings. In Sect. "Results", I present the results of modeling for EQ-5D-5L data. In Sect. "Discussion", the findings are discussed, also in the context of normative assumption underpinning the proposed solution. Brief conclusions end the paper in Sect. "Conclusion". In the Appendix, I included additional elements which are referred to in the main text.

## The problem and an idea of a solution

### The problem

Imagine a micro-society comprising of two friends: Rachel and Ross. Both are asked for their views on health, using the EQ-5D-5L descriptive system and cTTO.

Rachel considers the pits state much worse than dead. Specifically, the disutility of the pits state is twice as large as the disutility of dead. The ‘twice as large’ designation is meaningful for disutilities in terms of individuals whose preferences are expressed with vNM utilities. It can be operationalized using years of life (as in TTO), probabilities (similarly to standard gamble, SG, elicitation method), willingness to pay, number of people to be affected (as in person trade-off method), or some other cardinal parameter. In utility terms, $u_{\textit{Rachel}}(\textit{pits})=-1$. In LT-TTO, this preference would be reflected by Rachel being indifferent between immediate death and a mixed profile of $(\textit{FH},10)$ followed by $(\textit{pits},10)$. Ross, in contrast, considers the dead state to be twice as bad than the pits state in terms of disutilities. Hence, $u_{\textit{Ross}}(\textit{pits})=0.5$. In TTO, it would be reflected by Ross’s indifference between (11111, 5) and $(\textit{pits},10)$.

Averaging the two elicited utility values yields $u_{\textit{society}}(\textit{pits})=\frac{-1+0.5}{2}=-0.25$; hence, the pits state would be valued as WTD, as $u_{\textit{society}}(dead)=0$, based on the averaging of the utilities of individuals, each anchored on the QALY scale. In the societal value set, the pits state and being dead are assigned different weights, even though there is a clear symmetry in terms of how these two health outcomes are valued by the two individuals. This result is obtained from treating the disutility of being dead as a common yardstick, relative to which the other disutilities are measured.

**Fig. 1:**
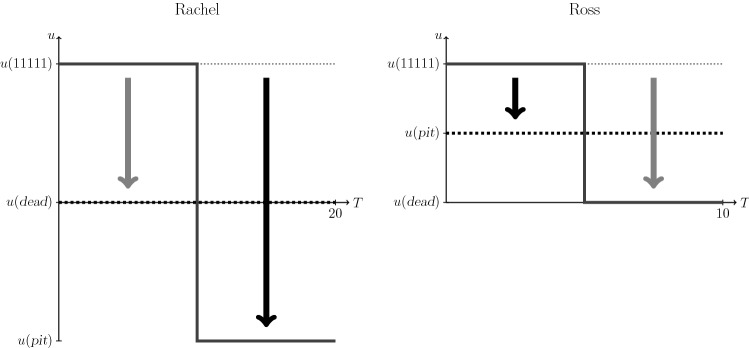
Preferences of Rachel and Ross for the pits state, as they would be illustrated in the time trade-off context. The axes were aligned to visually equate the position of dead and full health. The thick arrows represent the disutilities relative to full health

The above considerations are illustrated in Fig. 1. For Rachel (left panel), a mixed profile (solid line) comprising equal times in 11111 and the pits state is equivalent to not living at all (black thick dashed line). The disutility of dead relative to 11111 is depicted with a thick gray arrow, and the disutility of the pits state with a thick black arrow. For Ross, 5 years in 11111 followed by death (solid line) is equivalent to 10 years in the pits state (black thick dashed line). The disutilities have precisely the opposite relative magnitudes to those in Rachel’s case.

### A solution

Setting $u(\textit{dead})=0$ is a common practice. It is convenient in CUA to avoid having to sum the infinitely long streams of the utility of being dead. It is also theoretically justified in the QALY model within the framework of [7]. I present arguments why it should be the case when representing the preferences of an individual in Appendix A.

Nevertheless, the fact that for each individual separately, it may be required to assign $u(\textit{dead})=0$, does not imply that the zeros define a common anchor point across individuals. In economics, interpersonal utility comparisons are viewed as problematic, if not simply forbidden. That same values represent the utility of person A consuming good *X* and person B consuming good *Y* (also allowing $X=Y$) does not imply that any quality of perception is equal between A and B. In the context of health preferences, even if the utility of being dead should be assigned 0 in the theoretical models, it does not imply that the attitude towards being dead (and the perceived loss as compared to living in FH) is identical for all individuals in any sense. In other words, there is no deeper meaning to the algebraic equality $u_{\textit{Rachel}}(\textit{dead})=0=u_{\textit{Ross}}(\textit{dead})$, as $u_{\textit{Rachel}}(\cdot )$ and $u_{\textit{Ross}}(\cdot )$ are two separate entities, coming from separate contexts.

Because the vNM utilities are cardinal, the relative position of utilities within a range defined by other utilities can be meaningfully compared. For instance, assume that the utility of *Z* is half-way between the utilities of *X* and *Y* for each of two individuals. Then both individuals will be indifferent between a certain *Z* and a 50:50 lottery between *X* and *Y*. Therefore, agreeing on two health states defining the common yardstick to be used for all individuals would allow anchoring and averaging of the utility functions. However, as I claim here, it is not obvious that it is the difference between full health and dead that should serve this purpose. Some individuals may consider permanent health disabilities are more important or that improving the HRQoL deserves more public resources (than life saving programs), whereas some individuals may feel exactly the opposite. In spite of this, in the standard approach to value set construction, because the $\delta (\textit{dead})$ is used as a common yardstick, the symmetry between the relative importance of improving HRQoL or prolonging life may be broken, as shown in the illustrative example of Rachel and Ross.

The effect of equating $u(\textit{dead})$ between individuals whose utilities are averaged, as illustrated in Fig. 1, is that individuals with a larger utility range (i.e. those caring about the HRQoL more) have more impact on the final averages. In the example, the average utility of the pits state is $-0.25<0$, which agrees with Rachel’s ranking of the pits state as WTD.

The solution I propose is based on the premise that value sets are predominantly used to prioritize health technologies. Hence, their role is to inform about the relative importance of various health gains, in particular the gains in longevity or HRQoL (or equivalently, relative importance of disutilities of various health outcomes, being dead included). To equate the impact of each individual on the final values, I equate the numerical value of maximal attainable health gain between the individuals (within a fixed time horizon, e.g. 1 year). This is effectively obtained by rescaling each individual’s disutilities for all possible health outcomes (dead included) within a unitary range. Such rescaled utilities are averaged or used for econometric modeling. The relative disutility of being dead in the unit interval is also calculated for each individual and then averaged. Eventually, for convenience in CUA, the final averages of scaled disutilities are scaled again to restore the disutility of dead as being equal to 1 (hence, utility of 0).

Let us apply the approach introduced to Rachel and Ross. For Rachel, the pits state defines the bound of the range. Hence, the disutility of 55555 is rescaled to 1: $\delta _{\textit{Rachel}}(\textit{pits})=1$. After rescaling, $\delta _{\textit{Rachel}}(\textit{dead})=0.5$. For Ross, the pits state is better than dead (BTD): hence, the state of being dead defines the range: $\delta _{\textit{Ross}}(\textit{dead})=1$, and $\delta _{\textit{Ross}}(\textit{pits})=0.5$. The societal averages are $\delta (\textit{pits})=0.75=\delta (\textit{dead})$. Rescaling to have $\delta (\textit{dead})=1$ requires multiplying by $0.75^{-1}$, so eventually both disutilities are equal to 1 and both utilities are equal to 0.

**Fig. 2:**
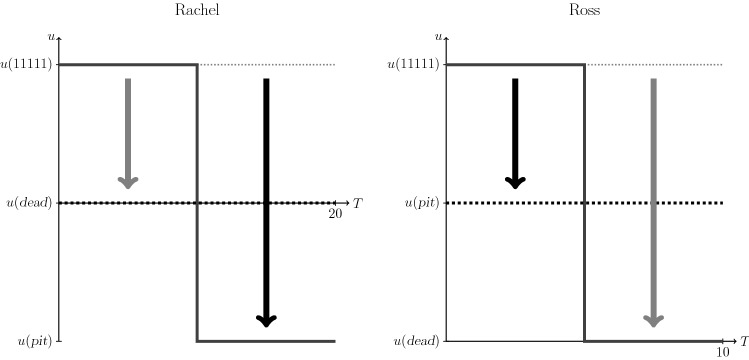
Preferences of Rachel and Ross as in Fig. 1 but here depicted to visually equate the largest possible improvement (equal to largest possible disutility), which restores the symmetry in relative preference for the improvements

The above approach is illustrated in Fig. 2. In this instance the axes were arranged to equate the largest possible improvement between the individuals, which restores the symmetry in relative views on the improvements between the states under consideration.

## Formal model specification

In the present section, I specify more formally the idea presented in subsection "A solution".

### A simplified econometric approach

Assume there are *N* individuals, indexed by $n\in \left\{ 1,\ldots ,N\right\}$. Each individual values $K_n$ states, denoted by $Q_{n,k}$, where $k\in \left\{ 1,\ldots ,K_n\right\}$ indexes the state. The utilities $u(Q_{n,k})$ are elicited on a QALY scale, with $u(dead)=0$ and $u(11111)=1$ for this individual. In what follows, I work with disutilities: $\delta (Q)=u(11111)-u(Q)=1-u(Q)$, with indices omitted here for brevity.

In the current subsection in the exposition of the model, I assume that *u*(*pits*) is observed and it is the lowest of values observed for each individual for all the health states studied. If this is not the case (for instance, due to data loss or random errors made by respondents in TTO), then a pragmatic solution that can be used in the modeling, is to use the actually reported worst state to define the largest disutility. The assumptions are relaxed in the Bayesian approach in the subsequent subsection.

I let *i* index the dimensions, $i\in \left\{ 1,\ldots ,5\right\}$, and *j* index the levels within the dimensions, $j=1,2,3$ for EQ-5D-3L or $j=1,\ldots ,5$ for EQ-5D-5L. Let $d_{i,j}(Q)$ be a dummy variable equal to 1 if in state *Q* the dimension *i* is at level *j*.

In the standard approach to value set construction, the following model is estimated

1 $$\begin{aligned} \delta (Q_{n,k})=\alpha _0 + \sum _{i,j} \alpha _{i,j} \times d_{i,j}(Q) + \varepsilon _{n,k}, \end{aligned}$$

with $\alpha _0$ occasionally being omitted (especially in EQ-5D-5L, see [13] for instance) and various approaches being used to specifying $\varepsilon$ (e.g., allowing its variance to differ between states or accounting for a panel structure of the data).

In the proposed approach, we rescale the disutility into a unit interval denoting maximal health gain by dividing the dependent variable by $\max (\delta (pits),1)$, for each individual. If all health states are valued as BTD, then being dead defines the largest disutility. To differentiate from the above standard approach, let us denote the model parameters by $\beta$. To rescale $\beta$s back in such a way that they are expressed on a scale for which the utility of being dead amounts to zero (for convenience in CUA), the results are divided by the scaling factor related to the average position of dead within the unit interval (the factor is calculated for the whole dataset, and not per individual):

2 $$\begin{aligned} D=N^{-1}\sum _{n=1}^N \min (1,\delta (pits)^{-1}). \end{aligned}$$

Because the model estimates are given by a linear function of the observed values of the dependent variable, both scalings can be done simultaneously by applying the scaling to the dependent variable, and the following model specification is used (indices defining individuals omitted for brevity):

3 $$\begin{aligned} \frac{\delta (Q)}{D\times \max (\delta (pits),1)}=\beta _0 + \sum _{i,j} \beta _{i,j} \times d_{i,j}(Q) + \varepsilon , \end{aligned}$$

with *D* as defined in Eq. 2 (i.e. a single value for all respondents).

In what follows, I use three approaches to defining $\varepsilon$, to see the impact of the proposed idea across various model specifications:

- ordinary least squares (OLS) — $\varepsilon _{n,k}$ is assumed to be identically and independently distributed for all *n* and *k*;
- generalized linear model (GLM) — $\varepsilon _{n,k}=\zeta _{n}+\eta _{n,k}$, i.e. there is a respondent-specific error term to capture the correlation of errors within a respondent;
- weighted least squares (WLS) — the variance of $\varepsilon$ is assumed to be different between states. To account for this, all of the variables (dependent and independent) are divided by the standard deviation of disutilities observed for each health state separately.

Even if the focus on this paper is not on fine-tuning the econometric approach, the multitude of specifications was used to show the range of impact of the scaling proposed in the paper.

### Bayesian estimation

This approach accounts for common issues with the data: missing values (also missing $u(\textit{pits})$), censoring (utilities $<-1$ not possible to be reported in the usual cTTO), and inconsistencies in observed values ($u(\textit{pits})$ not being the lowest value). In the Bayesian approach, we specify the *a priori* assumptions regarding model parameters and how the observed values depend on these parameters. I present the model for the EQ-5D-3L case, while EQ-5D-5L differs only slightly by having additional levels. Here, the outline of the model is presented, while interested readers may refer to Appendix B for further details (the actual JAGS code). In the Bayesian estimation, the following elements are specified:

- the pits state disutility (differs between individuals) and the implied relative dead position,
- the relative importance of dimensions at their worst levels (assumed to be identical between individuals),
- the relative importance of levels within each dimension (assumed to be identical between individuals),
- how the actually observed utilities are distributed around their expected values.

The pits state disutility, as measured on the QALY scale, is drawn for each respondent from a log-norm distribution, with parameters endowed with non-informative priors, $\delta _{QALY}\sim \textit{log-norm}$. The dimension importance at the worst level (i.e. MO3, SC3, ...) and a constant are taken to be identical across respondents and are drawn from a Dirichlet distribution to sum up to 1. The importance of level 2 relative to level 3 is a parameter with a uniform prior on [0, 1]. In consequence, the relative importance of all EQ-5D health states is identical across the respondents, and only the position of dead is assumed different.

The above model has been used both with and without the proposed modification (i.e. within-individual rescaling), to capture the impact of the rescaling on the results. In the standard approach (without the rescaling), the parameters measuring the disutilities for dimensions/levels are multiplied by $\delta _{QALY}$ to calculate the disutility for a given state on the QALY scale.

In the proposed approach with rescaling, the relative position of dead on the QALY scale is calculated for each respondent as $\max (1,\delta _{QALY})^{-1}$. The average of these individual values is calculated to be used as a scaling factor to arrive at values convenient for CUA, i.e. with the final utility of dead equal to 0. One more correction is required: for individuals valuing the pits state as BTD, the relative dimension importance should be measured relative to the disutility of dead (and so should sum to $<1$). Therefore, the average of the following terms is calculated so as to correct the dimension importance downwards: $\min (1,\delta _{QALY})^{-1}$.

I assume that the disutility of a given health state (which implies the outcome of the TTO task) is randomly drawn around the average calculated based on $\delta _{QALY}$ (idiosyncratic), the dimension importance, and the relative level weight. I assume there is censoring for disutility values greater than 1.975, the standard deviation of the error depends proportionally on the average disutility, and the distribution of errors is given by a generalized t-distribution, with the numbers of the degrees of freedom to be estimated (to allow for fat tails).

To additionally test the robustness of the methods and results, I accounted for the possibility of a non-linear time preference, as this possibility is recently more and more widely advocated and used in the literature [18, 19]. I assumed power discounting with an idiosyncratic discount parameter, in view of the large heterogeneity [20]. Because estimation of this extended model entails additional parameters, this was only done for the larger EQ-5D-5L dataset.

The model was estimated in Jags [21], with 4 chains and 1000, 10000, and 5000 iterations for adaptation, burn-in, and actual calculation, respectively. No problems with convergence were observed (by monitoring potential scale reduction factors).

## Results

I applied the proposed methods to two datasets collected in Poland, in the only EQ-5D-3L and EQ-5D-5L valuation studies conducted there. More details on the data collection can be found in the original valuation publications [13, 16]. The EQ-5D-3L study used the older version of TTO in which for WTD states, very low values (even $-39$) were elicited. Due to lack of face validity, these values were commonly transformed with a rather arbitrary transformation to fit into $(-1,0)$ interval, which makes the negative values dubious. Additionally, in the EQ-5D-3L valuation, a small sample of 321 respondents was recruited. Therefore, the results for this dataset are delegated to Appendix C, and only the results for the EQ-5D-5L are presented in the main text.

In the EQ-5D-5L study conducted in 2016, a much larger sample of 1252 respondents was used, representative in terms of age, sex, education, region, and size of locality. Apart from demographic questions, warm-up tasks, and DCE tasks, each respondent valued 10 hypothetical health states with cTTO during an computer-assisted personal interviews.

Barely 21 respondents ($<2{\%}$) did not value the pits state as the worst one. In those few respondents, the mean and median of the excessive disutility of other-than-pits state amounted to 0.24 and 0.2, respectively. In the econometric analysis, the rescaling was done using the largest disutility reported. There were 63% of respondents who valued the worst health state as strictly worse than dead, and 6.6% exactly at 0. In 96.1% of the respondents, the pits state (55555) value was available. The average scaled disutility of dead amounts to 0.728.

The results of the modeling are presented in Table 1. No intercept was used, as the enhanced level structure typically leads to its insignificance. The results are very similar to the findings in the original valuation study [13], including a slight inconsistency of parameters in some specifications (MO2 and MO3 ordering, see Table 2 of the referenced paper). However, the final value set in [13] also used the DCE data in a hybrid approach.

**Table 1:** Results for EQ-5D-5L. In subsequent parts: disutilities for dimensions/levels; utilities for 22222, 33333, 44444, and 55555; % of health states with negative utility; relative importance of individual dimensions (level 5 in a single dimension divided by level 5 values summed across all the dimensions)

Param.	OLS	OLS*	GLM	GLM*	WLS	WLS*	Bayes	Bayes*	Disc	Disc*
MO2	0.021	0.018	0.022	0.019	0.031	0.030	0.015	0.012	0.028	0.024
MO3	0.012	0.009	0.012	0.011	0.028	0.021	0.074	0.006	0.026	0.022
MO4	0.099	0.093	0.098	0.091	0.110	0.101	0.090	0.069	0.107	0.089
MO5	0.262	0.222	0.262	0.222	0.270	0.233	0.219	0.167	0.246	0.204
SC2	0.037	0.036	0.031	0.027	0.032	0.033	0.023	0.017	0.035	0.029
SC3	0.043	0.039	0.038	0.035	0.037	0.031	0.037	0.028	0.063	0.053
SC4	0.117	0.111	0.122	0.107	0.105	0.098	0.114	0.087	0.124	0.103
SC5	0.271	0.231	0.277	0.234	0.259	0.219	0.269	0.205	0.299	0.248
UA2	0.035	0.028	0.033	0.026	0.027	0.027	0.015	0.012	0.024	0.020
UA3	0.042	0.035	0.033	0.030	0.042	0.037	0.027	0.021	0.034	0.028
UA4	0.090	0.080	0.093	0.086	0.093	0.081	0.110	0.084	0.131	0.109
UA5	0.185	0.165	0.187	0.163	0.186	0.168	0.202	0.154	0.213	0.177
PD2	0.033	0.033	0.029	0.029	0.029	0.030	0.018	0.014	0.028	0.024
PD3	0.035	0.039	0.034	0.038	0.039	0.038	0.044	0.033	0.074	0.062
PD4	0.228	0.216	0.229	0.213	0.238	0.218	0.237	0.181	0.261	0.217
PD5	0.472	0.425	0.468	0.423	0.486	0.435	0.535	0.408	0.506	0.421
AD2	0.033	0.026	0.025	0.023	0.019	0.020	0.012	0.009	0.019	0.016
AD3	0.033	0.025	0.035	0.032	0.025	0.024	0.019	0.014	0.025	0.021
AD4	0.114	0.105	0.116	0.108	0.109	0.102	0.108	0.082	0.129	0.107
AD5	0.228	0.201	0.225	0.202	0.212	0.194	0.225	0.172	0.243	0.202
22222	0.841	0.860	0.860	0.875	0.862	0.860	0.917	0.937	0.870	0.883
33333	0.834	0.853	0.848	0.855	0.829	0.850	0.799	0.898	0.782	0.810
44444	0.352	0.396	0.342	0.395	0.345	0.401	0.342	0.498	0.266	0.357
55555	$-0.418$	$-0.245$	$-0.418$	$-0.245$	$-0.412$	$-0.248$	$-0.449$	$-0.106$	$-0.465$	$-0.294$
% $u<0$	2.9%	0.8%	2.8%	0.8%	2.8%	0.8%	4.0%	0.1%	4.3%	1.2%
MO	18.5%	17.8%	18.5%	17.8%	19.1%	18.7%	15.1%	15.1%	13.9%	19.0%
SC	19.1%	18.6%	19.5%	18.8%	18.3%	17.5%	18.6%	18.5%	20.4%	19.2%
UA	13.0%	13.3%	13.2%	13.1%	13.2%	13.5%	13.9%	13.9%	14.5%	13.7%
PD	33.3%	34.2%	33.0%	34.0%	34.4%	34.8%	36.9%	36.9%	34.5%	32.5%
AD	16.1%	16.2%	15.9%	16.2%	15.0%	15.5%	15.5%	15.6%	16.6%	15.6%

Scaled models are denoted with an asterisk. *OLS* ordinary least squares, *GLM* generalized linear model (individual error term), *WLS* weighted least squares, *disc* Bayes with discounting

For all the modeling specifications, the scaling proposed in this paper reduces the disutilities assigned to individual dimensions/levels and as a result it increases the estimated utilities of health states. The relative importance of dimensions barely changes with the proposed rescaling approach.

Interestingly, the Bayesian approach (without discounting) decreases the (negative) utility of 55555 for the standard approach, i.e. without scaling, and increases it (making it less negative) for the proposed scaling. As a consequence, the net impact of scaling is largest in this modeling approach. The proportion of negative value health states decreases with scaling, from about 3% to 4% to less than 1%, or even 0.1% in the Bayesian approach.

Accounting for the possibility of discounting attenuates the impact of rescaling, but the changes are not substantial. The results of the Bayesian estimation with discounting suffer from the aforementioned slight inconsistency of parameters (MO2 and MO3).

## Discussion

### Normative aspects of comparing utilities between individuals

In economics, interpersonal utility comparisons are viewed as problematic, if not simply forbidden. How value sets for health states are currently constructed suggests that this fact is largely disregarded in health preference research.

Admittedly, the value sets are needed to support CUA and, therefore, aggregating in some way the preferences of the members of the society is needed. Hence, it may be necessary to develop a list of properties that the method of anchoring of the values between individuals should have. What properties are selected is a normative and arbitrary decision. Hence, developing a list of properties that would be widely accepted seems an important direction for further research. Below, I present what properties the method proposed in the present paper has.

A rather obvious property to consider is the *Pareto-rule*. If every member of the society prefers one health outcome to the other, the former should be assigned greater value in the value set. It is rather obvious to notice that both the standard approach and the proposed approach have this property (the rescaling does not change the ordering of the health outcomes—including being dead—hence, the final ordering is identical). Interestingly, it is possible for some reasonable ways of preference aggregation to violate the Pareto-rule (see an example in Sect. "Relation to other research" below).

The standard and the proposed methods satisfy a stronger version of the Pareto-rule that I call *preservation of relative preference unanimity*. If every member of the society considers the disutility of one health outcome, $q_1$, to be $\lambda$ times larger than the disutility of some other health outcome, $q_2$, then in the final value set the same relative relation should hold. The property holds in the proposed method, because rescaling does not change the relative disutilities between any two health outcomes (including being dead).

Now, I introduce another property, referred to as *relative preference inversion*, that is central to the main example of Rachel and Ross in Sect. "The problem" and that differentiates the standard and the proposed approach (and which is inspired by property 5 of the social welfare function presented in [22]). Consider the relative disutilities of pits state and being dead in members of the society (possibly different between individuals), i.e. $\nicefrac {\delta _i(\textit{pits})}{\delta _i(\textit{dead})}=\lambda _i$, where *i* indexes the individuals, $i=1,\ldots ,N$ (which simplifies in the QALY scale where $\delta _i(\textit{dead})=1$). Measure the relative disutility in the aggregate value set. Now, assume each individual inverts their relative preference, i.e. changes $\lambda _i$ to $\lambda _i^{-1}$. Then, the relative disutility in the aggregate value set should also be inverted.

The main example clearly demonstrates that the standard approach violates the relative preference inversion property. Inverting the preferences effectively swaps the preferences between Rachel and Ross. Hence, it has no consequence on the final value set, and so the aggregate disutility is not inverted. Meanwhile, it can rather easily be shown that for the proposed method the relative preference inversion property holds. The aggregated rescaled disutilities of pits would amount to $N^{-1}\times \sum _{i,1\le i\le N}\min (\lambda _i,1)$ and to express the final values on the scale with disutility of dead equal to 1, it would be divided by the *D* factor (see Eq. 2) equal to $N^{-1}\times \sum _{i,1\le i\le N}\min (\lambda _i^{-1},1)$. Now, obviously the result is inverted if $\lambda _i$ are inverted.

There is another normative appeal of the proposed approach: it is more egalitarian than the standard approach as illustrated in the following example. Imagine that Rachel or Ross, *ceteris paribus*, re-evaluate the pits state and increase its disutility relative to FH by 10% of the original value. Because the absolute values of utility functions in principle are meaningless, I find considering the relative change more justifiable. Hence, if Rachel re-evaluates the pits state, we have $u_{\textit{Rachel}}(\textit{pits})=-1.2$ (i.e. $\delta _{\textit{Rachel}}(\textit{pits})=2.2$, assuming it can be measured in TTO without censoring at $-1$) and $u_{\textit{Ross}}(\textit{pits})=0.5$. If Ross re-evaluates the pits state, we have $u_{\textit{Rachel}}(\textit{pits})=-1$ and $u_{\textit{Ross}}(\textit{pits})=0.45$. In the standard approach, the final value of the pits state would be $-0.35$ in the former case or $-0.275$ in the latter case, i.e. the difference from the staring case would be either 0.1 or 0.025 depending on who re-evaluates the pits state. In the proposed approach, the final value of the pits state would be almost identical for both cases, i.e. approx. $-0.031$ or $-0.033$. Hence, it would barely matter who re-evaluates the pits state, which demonstrates that the proposed approach makes the impact of individuals more equal.

It is worth noticing that the violation of the preference inversion symmetry by the standard approach cannot simply by explained and justified by the utility of health states having no lower bound in the QALY model. It is true that we might enlarge the descriptive system and keep on adding worse and worse states (admittedly this would be limited by the ability of inventing suitable adjectives). However, I contend that this does not invalidate the arguments presented in this paper, for several reasons. First, the fact that new alternatives can be added does not address the problems with interpersonal utility comparisons, i.e., it does not imply that the perceived difference between dead and 11111 is the same across individuals. Second, the current paper refers to the situation when actual values for a given descriptive system have already been collected, i.e. we consider a fixed set of numbers (and so the potential unboundedness is beyond the scope). Finally, the proposed method does account for the possibility that the pits state may be valued arbitrarily low by any individual (or that new pits states can be added to the descriptive system that are even worse). For instance, if Rachel or Ross re-evaluate the pits state and consider it is worse (relative to how bad being dead is) than previously, then the resulting utility of pits would decrease as well (as the example in the preceding paragraph shows).

The rescaling proposed in the present paper focused on the bottom part of the utility scale, i.e. the pits state or death, whichever was deemed worse by the respondent. I believe that no corresponding dilemmas need to be considered for the upper part of the scale. First, it seems a safe assumption that 11111 health state (in EQ-5D-3L or EQ-5D-5L) will be considered as the best alternative. Therefore, no alternative states are available to define the upper bound of the utility range for an individual. Obviously, interpersonal comparisons caveat applies, i.e. the joy of being in 11111 cannot be compared across individuals in any economically warranted way. Nevertheless, what values are assigned to *u*(11111) across individuals (and whether $u(11111)=1$ for everyone) is unimportant, as it is only the disutility, i.e. the difference between the 11111 and a given state, that matters for the calculations.

### The results and their implications

The result of the proposed approach both in EQ-5D-5L and EQ-5D-3L case was to increase the final values for all health states. Obviously, the approach proposed would have no impact, if everyone valued all states as BTD. If all respondents valued pits state as WTD, there still would be no impact if all respondents valued the pits at exactly the same level. In a more realistic case that the respondents are heterogeneous (still assuming all value pits as WTD), the difference between the final value of the pits state in the value set would correspond to the difference between arithmetic and harmonic means. To see this, denote by $\delta _i(\textit{pits})>1$ the disutility of pits by *i*-th respondent (on the QALY scale). The final value resulting from the standard approach is $N^{-1}\sum _{i}\delta _i(\textit{pits})$. In the proposed approach, the pit state would define the unit range for all the respondents. Hence, the final value resulting from the proposed approach is the inverse of *D* (Eq. 2), i.e. $\nicefrac {N}{\sum _{i}\delta _i^{-1}(\textit{pits})}$.

Let us consider a simplified case of only two respondents, who value the disutility of pits at $\delta$ on average, but their individual disutilities differ and amount to $\delta -\Delta >1$ and $\delta +\Delta$, for some $\Delta \ge 0$ representing heterogeneity (equal to the standard deviation of disutilities). It can be easily calculated that the absolute difference between the standard and the proposed approach would amount to $\nicefrac {\Delta ^2}{\delta }$, and the difference relative to $\delta$ (i.e. the outcome of the standard approach) would amount to $\left( \nicefrac {\Delta }{\delta }\right) ^2$, i.e. the squared variability coefficient of the individual pits disutilities. This result suggests that the impact of the proposed approach increases with the heterogeneity of the respondents.

Coming back to the empirical results in EQ-5D-5L and EQ-5D-3L, the effect of using the rescaling and anchoring proposed in this paper is much stronger in the Bayesian approach to estimation. This is most likely caused by accounting for censoring in this approach. In this case, the observed utilities of $-1$ are treated as possibly $<-1$, which gives room for even stronger scaling.

When accounting for possible discounting with discount rates across individuals, the impact of the proposed approach to scaling/anchoring diminished yet remained important: scaling increases the utility of the pits state by more than 0.15. Assuming a constant discount rate across the respondents (not reported in the paper) makes the impact of discounting much smaller. In view of the difficulty of estimating the respondent-specific discount rate indirectly, i.e. from TTO tasks, instead of using some direct approach (e.g., [23]), I think more caution is required when interpreting the results obtained with discounting, both in terms of its impact on the scaling and the actual disutilities assigned to dimensions/levels.

The comparison of the results between EQ-5D-3L and EQ-5D-5L is difficult for at least three reasons. First, the descriptive systems changed. Therefore, even the same wording of the levels when put in the context of other surrounding levels might be interpreted differently by the respondents. Second, the preferences of Poles might have changed over the decade that separated the studies, and the first sample was much smaller and representative only with respect to age and sex, but not education or geographical region. Most importantly, however, the EQ-5D-3L valuation study used the previous TTO protocol, in which the elicitation of utilities for WTD states allowed for very negative values to be elicited. This negative utilities were arbitrarily rescaled to fit into a $[-1,0]$ interval, as was commonly done in valuation studies at that time. I decided not to correct back for this rescaling in the present paper, in order to show the impact of the proposed rescaling for the utility values as they were elicited and used in the original study.

Clearly, the properties of the proposed approach depend on the descriptive systems used. What the pits state is and as how bad it may be perceived (relative to dead) by an individual heavily depends on the definition of dimensions and levels. It seems that as progressively worse levels are included (and the more dimensions there are), the worse the pits state may be valued by most. Consequently, the impact of the proposed approach as compared with the standard one will increase.

Additionally, the impact of the proposed approach on the utility values will be larger in societies who are more willing to accept some states as WTD. In the Polish context, it was shown that religiosity is associated with non-trading and not accepting that a state is WTD [24]. As Poland is still more religious than most of the European countries, the effects of the proposed methodology may be larger in other societies.

The effect of the proposed scaling in CUA would be to increase the priority of life prolonging treatments. This would happen via two mechanisms. First, the improvements in longevity would be multiplied by higher on average utilities of states, which would generate more QALYs with the same number or extra years of life. Second, the improvements of quality would generate fewer QALYs, which would make HRQoL-improving interventions relatively less attractive. Particularly in the Bayesian approach in EQ-5D-5L (Table 1), the value set built with the scaling proposed in this paper has barely any states with negative utilities. Hence, life prolonging is beneficial in virtually all settings (though clearly not necessarily cost-effective), which—as a by-product—alleviates some doubts raised recently about negative utilities [25]. The resulting shift in decisions would be favored by respondents who barely trade in TTO (or perhaps even demonstrate lexicographic preferences) and are assigned high utilities to health states in result.

Using other model specifications (e.g. accounting for respondents heterogeneity in Bayesian approach) could change the results. However, the present paper focuses on a more fundamental issue of interpersonal utility comparisons than on selecting a specific econometric approach. In any case, several methods of data analysis were presented in the current paper to demonstrate the robustness of the overall results of the paper.

### Relevance to other types of tasks used in valuation of health states

In the paper, I only considered those utilities elicited with TTO. I believe that the same issue can be identified in the SG method, as the difference between TTO and SG only lies in what is used to obtain the cardinal utilities in the elicitation task (duration in TTO, probability in SG). A person may only accept a small probability of death in SG, because they highly value a given health state or because they have a great fear of death. Hence, in SG the interpersonal utility comparisons are also a problem.

Perhaps ironically, the problems with interpersonal comparisons discussed in this paper do not affect the visual analogue scale (VAS, see [26] for more information on this method), a method of valuing states that is otherwise criticized for lack of theoretical foundations. VAS was mostly employed in the past (e.g., [27–29]). In VAS, the respondents are asked to assign to each health state a value on the 0–100 scale, where 0 is said to denote the worst possible health state. Additionally, respondents are typically asked to mark where dead is on this scale. VAS not being a choice-based task hinders the interpretation of the resulting numbers. What is interesting in VAS, in the context of the present paper, is that each respondent is forced onto the same 0–100 scale. Hence, applying the approach proposed in this paper for VAS, would require no prior rescaling of values coming from individual respondents (only averaging and then rescaling the averages to obtain the utility of dead equal to zero for convenience). Obviously, the values resulting from such a procedure would still lack theoretical foundations as they would not come from a preference-based elicitation technique.

DCE (see [30] for more information) suggests a means by which the proposed approach could be tested. In TTO, Rachel and Ross in the example can be claimed to be fully symmetrical *a priori* (with the relative importance of dead and pits state improvements reversed), and it was the arbitrary selection of the yardstick that broke this symmetry. In DCE, however, if we assume the same scale factor between the individuals, the ability to repeat the measurement in both individuals, and the independent random terms between these repetitions, then new insight can be gained. For instance, assume the frequency with which Rachel chooses dead over the pits state amounts to 90%, and the frequency with which Ross chooses the pits state over dead amounts to 70%. Then we may say the difference between the two states is perceived to be larger by Rachel than Ross. I believe this may be one of the directions for further research in the context of problems highlighted in the present paper, but the heterogeneity of respondents with respect to scale would have to be controlled for.

### Relation to other research

First, let us notice that the idea of considering the preferences within ranges defined on case-by-case basis is making its way into decision theory. In [31], it is shown how analyzing utility in the context of choice under risk with monetary pay-offs in ranges defined by lotteries available may improve the prediction capability and explain a selection of paradoxes.

In the health context, a similar strand of research to the one in the current paper was started independently [32]. In this study, the authors also identified the problem of interpersonal utility comparison when calculating value sets, yet their approach differs in one important factor. They propose scaling the disutilities elicited by individuals in a [0, 1] range, with the bottom always defined by the pits state (ignoring how it relates to the dead state). Then the disutilities for health states are averaged, and the relative position of the disutilities to the dead is calculated by the average range of disutilities between individuals. To put it differently, the averaging of within-HRQoL preferences and HRQoL-vs-longevity preferences is done separately. There are several consequences of this, as outlined below.

The change to the value set with the approach presented in [32] is much smaller, since it only corrects for the possible differences in the relative dimension importance between respondents who are reluctant to trade and those willing to trade a lot. In contrast, the approach in the present paper impacts the relative value of longevity gains vs HRQoL gains, and the relative importance of the dimensions was shown to change very little.

The authors in [32] seem not to treat dead as a state, while in CUA it is often an outcome (to be avoided by using a health technology). From a technical point of view, the approach in [32] is problematic for total non-traders (i.e. respondents who traded in no TTO task). Then, no meaningful rescaling can be performed using the approach presented in [32], while the present approach would easily rescale the utilities in the interval having its bottom defined by dead.

Interestingly, the rescaling only relative to pits as in [32], violates the Pareto-rule as defined in the beginning of the Discussion. Notice the following example. Consider a microsociety of two people—Alice and Bob—who value two health states: a moderate state and pits state. The obtained LT-TTO utilities are the following: $u_\textit{Alice}(\textit{moderate})=0.1$, $u_\textit{Alice}(\textit{pits})=0.05$, $u_\textit{Bob}(\textit{moderate})=0.1$, and $u_\textit{Bob}(\textit{pits})=-1$. Both individuals consider moderate state BTD. The relative disutility of moderate state to pits state amounts to approx. 0.947 for Alice and to 0.45 for Bob, i.e. approx. 0.699 on average. The average pits state disutility amounts to 1.475. Combining the two averages would yield the aggregate disutility of moderate of $0.699\times 1.475\approx 1.031$, which would make this state WTD.

The treatment of dead as any other state makes the current paper quite different from the perspective taken in a recent study [33]. I agree with the authors that the presence of dead in valuation studies may make the process difficult for some respondents and might impact the results (Section 6 of the referenced paper). One might also argue that dead is qualitatively different from other health states: it surely also deprives someone of other beyond-health forms of quality of life. On the other hand, when valuing health states, individuals most likely account for their specific beyond-health situation, e.g. having someone to help them with their usual activities. Most importantly, prolonging life (hence, postponing death) is why most health technologies are used, and the effect thereof needs to be quantified in CUA. Therefore, dead needs to taken into consideration to measure health gains relative to it.

From a more pragmatic perspective, that being dead (immediate death) is explicitly used in the currently employed elicitation methods is a fact. In TTO, some questions directly ask respondents to compare some living state with immediate death. In DCE, even if a version with duration is used, it is often enhanced by some comparisons vs dead (see [34], for example). The present paper focuses on how available data/approaches should be modified to account for restrictions in interpersonal utility comparisons.

I also think that the distinction between BTD and WTD states is rather natural and well defined. It may be understood as the difference between the states in which the prolongation of life is desired and the states in which it is the shortening that is wanted. Therefore, the negative values for some states arise quite naturally to represent utility decreasing with duration. Nonetheless, in some contexts, it may be found as a convenient fact that the approach proposed here reduces the proportion of states treated as WTD in the resulting value set [25].

Finally, in a recent study ( [35], henceforth, DT) the issue of recalculating utilities between various scales and averaging the utilities between groups of people is also discussed. However, the motivation of DT is quite different from the present paper, and their approach is in some sense precisely the opposite. DT consider the problem of moving between two utility functions: $u(\cdot )$, with $u(\textit{dead})=0$, and $u'(\cdot )$, with $u'(\textit{pits})=0$ (denoted as AW, *all-worst*, in their paper). They assume $u(\cdot )$ is available for people considering the pits state as BTD, and $u'(\cdot )$ is available for people considering the pits state as WTD. DT then show that the two following approaches yield different results: (i) whether $u(\cdot )$ is rescaled onto $u'(\cdot )$, then averaged across all individuals, and finally rescaled back onto $u(\cdot )$, or (ii) $u'(\cdot )$ is rescaled on $u(\cdot )$ and then averaged.

There are at least four vital differences between DT’s and the present paper. The first difference is that the motivation of DT does not stem from interpersonal utility comparisons limitation; instead, it seems more of a purely algebraic or technical nature. Second, DT consider the situations in which two scales are used simultaneously for subgroups of a single group of respondents, depending on the preferences of individual respondents (i.e. the pits state being BTD or WTD). Meanwhile, in TTO, all the utilities are elicited on a scale with $u(\textit{dead})=0$ for each individual. Hence, the problem considered by DT does not prevail.

The third difference is that DT’s results can be interpreted to show that the selection of the common-yardstick changes the result. For instance, in Eq. 4 of DT, the utility of dead is averaged between the two subgroups of respondents, first rescaling $u(\cdot )$ into $u'(\cdot )$. Hence, the difference between the pits state and full health is essentially treated as a common yardstick. In Eq. 6 of DT, the utility of the pits state is averaged, rescaling $u'(\cdot )$ into $u(\cdot )$. Hence, the difference between dead and full health is treated as a common yardstick. Meanwhile, in the present paper, quite the opposite procedure is employed. I claim that using any of the above differences in utilities is unfair, since it is based on interpersonal utility comparisons. Instead, I propose rescaling the utilities within an individual respondent. According to the DT notation, I start with $u(\cdot )$ and propose using $u'(\cdot )$ for people considering the pits state as WTD, to force all the utilities on the [0, 1] interval to distribute the impact on utilities more equally.

Finally, DT focus on averaging the utilities between the two subgroups of respondents. Meanwhile, the problem of scaling also arises when all the respondents value the pits state as WTD, as shown in the following example. Consider another micro-society, consisting of Tracy and Ted. Tracy values the pits state as equal to dead, and Ted values it as considerably worse than dead (and here I assume the implementation of TTO that allows eliciting very negative utilities). Assume $u_{\textit{Tracy}}(\textit{pits})=0$ and $u_{\textit{Ted}}(\textit{pits})=-10$, with the standard $u_{\textit{Tracy}}(\textit{dead})=u_{\textit{Ted}}(\textit{dead})=0$. The standard averaging yields the societal value $u(\textit{pits})=-5$. Observe that it is much closer to Ted’s preference in the following way. Tracy considers the improvements from the pits state to full health and from dead to full health as equally important. To Ted, the improvement from dead to full health is negligible in relation to the improvement from the pits state to full health. Putting it differently, the relative disutility of dead to the disutility of pits amounts to 100% and approx. 10% (i.e. $\nicefrac {1}{11}$) for Tracy and Ted, respectively. After standard averaging, it amounts to approx. 17% (i.e. $\nicefrac {1}{6}$), i.e. much closer to 10%. It can be easily verified that with the approach proposed in the present paper we would eventually get $u(\textit{pits})=-0.8{\overline{3}}$, and so the relative disutility amounts to approx. 55%.

## Conclusion

Setting priorities between health technologies improving either longevity or quality of life requires careful assessment of which of these is perceived as more important by society. The currently used methods for building value sets ignore the interpersonal utility comparison problem. They treat the utility difference between being dead and full health as a common yardstick between individuals to average preferences. As I show, it may unfairly grant more impact on the final value set to people favoring HRQoL gains. I show how to correct for this bias. In general, switching between individual and societal utilities must be handled with care. More awareness is needed with respect to the normative assumptions behind the currently used method and more discussion is needed on how to aggregate individual preferences for health.

## A Zeroing in on dead in utility elicitation

Since assigning 0 as the utility of dead and aligning all other utilities by equating these zeroes between the individuals is the source of the problem raised in the paper, it is useful to discuss the foundations of how utilities are assigned to health states and in what sense must dead have zero utility.

### A.1 Constant health profiles

In [7], the foundations for the utility representation of the preferences in the QALY model with chronic profiles (i.e. when health state does not change until death) were laid out. A single decision maker is considered. She has preferences over the set of possible (*Q*, *T*), where *Q* denotes a state and *T* denotes a duration, after which death occurs. In such a system, (*Q*, 0) effectively represents immediate death.

Standard vNM assumptions are used for the preferences over profiles. In consequence, the preferences can be represented by *v*(*Q*, *T*), i.e. $(Q_1,T_1)\succeq (Q_2,T_2) \Leftrightarrow v(Q_1,T_1)\ge v(Q_2,T_2)$. In [7], a set of assumptions under which $v(\cdot ,\cdot )$ can be decomposed into the impact of state and duration separately (also, see [8]). Briefly: assuming risk neutrality wrt. *T*, $v(\cdot ,\cdot )$ can be represented as $v(Q,T)=g(Q) + u(Q)\times T$. Further, the zero condition implies that *g*(*Q*) must be a constant for all *Q*, say $g(Q)\equiv g$. For convenience, it is usually assumed that $g=0$; then, the total utility of immediate death (i.e. $T=0$) is zero. However, $g=0$ is not a necessary condition for the formula to represent preferences. Furthermore, the value *g* is added to all health profiles (also when $T>0$). Therefore, it must not be interpreted as the utility of immediate death but — perhaps — as the utility of dying eventually, present in all the profiles.

If $\theta$ represents (in this subsection only) the state of being dead (or is preferentially equivalent), then it must hold that $(\theta ,T) \sim (Q,0)$, for any *T* and *Q*. Therefore, $g + u(\theta )\times T = g + u(Q)\times 0\Rightarrow u(\theta )=0$. In this sense, the model implies that the utility of dead (treated as a state that can last) must be equal to zero.

It may be useful to clarify that this necessity does not contradict the general possibility of affine transformation of the vNM utility. It is the function $v(\cdot ,\cdot )$ that represents the vNM utility, while $u(\cdot )$ is just one component in *v*’s algebraic representation. A constant may be added to $v(\cdot ,\cdot )$, which effectively changes *g*; $v(\cdot ,\cdot )$ may be multiplied by a strictly positive constant, which would rescale *g* and $u(\cdot )$.

### A.2 Non-chronic profiles

Let us now consider the possibility of the health state changing over time in discrete sub-periods, followed by death. The set of available alternatives is defined as $X=\bigcup _{T=0}^{+\infty }Q^T$, where *Q* is a set of possible one-period health states. It is $\emptyset \in X$, which represents immediate death.

Conditions have been given in the literature for the preferences over non-chronic profiles to be represented by an additive utility function [36, 37]. I assume that for ${\mathbf {x}}\in X$, the utility is given by $u({\mathbf {x}})=\sum _{T=0}^{dim({\mathbf {x}})} v(x_T)$, where $dim(\cdot )$ denotes the length of the vector, i.e. the duration of the health profile, and $v(\cdot )$ denotes the one period utility function. If $dim({\mathbf {x}})=0$, the sum is defined to amount to 0.

The preferences could be equivalently represented by $u({\mathbf {x}})=\sum _{T=0}^{dim({\mathbf {x}})} v(x_T)+g$, for any *g*. Hence, immediate death is not necessarily assigned zero utility. However, consider expanding *Q* by adding an element $\theta$ which represents (in this subsection only) being dead in a given period. For logical plausibility, we may only consider ${\mathbf {x}}$ such that if $x_i=\theta$ then $x_j=\theta$, for any $i<j\le dim({\mathbf {x}})$. By definition, ${\mathbf {x}} \sim [{\mathbf {x}},\theta ]$, i.e. explicitly adding a single period of being dead to the profile does not alter its attractiveness. This fact directly implies $v(\theta )=0$. Therefore, the conclusion drawn previously in the constant profile case holds.

### A.3 A pragmatic approach to cost-utility analysis

Because the assignment of utilities to health states is mostly done in order to subsequently use them in CUAs, it is reasonable to consider the situation resembling the construction of such models. A time horizon is typically pre-specified, $T>0$. Here, discrete time is assumed, i.e. $T\in {\mathbb {N}}$. We consider $X=Q^T$, where *Q* is a set of one period states. The total utility accrued is represented, ignoring discounting, by $\sum _{t=0}^{T}v(x_t)$. Being dead is explicitly accounted for as a state, e.g. in Markov models, $\theta \in Q$. Typically, a difference between the total effects in two arms, i.e. two vectors: ${\mathbf {x}}$ and ${\mathbf {y}}$, is calculated. Notice that when $v(\cdot )$ is modified additively by a constant *g* this does not change the difference. In this sense, in the present approach the one-period utility of dead may be non-zero.^1^

Summing up the above considerations: the utility of being dead (as a lasting state) must be zero in the QALY model when the time horizon may change for both chronic/non-chronic profiles, to ensure logical consistency. This is not to say that the utility of immediate death must be zero. Importantly, all the considerations are done within a single individual whose preferences are being modeled and represented with the utility function. The algebraic necessity within each individual does not imply that the perception of this state is equal between individuals. In CUA, if the time horizon is fixed and the focus is on comparisons of utilities accrued between various scenarios, the dead state utility may be non-zero without affecting the results.

## C Results for the EQ-5D-3L data

In the original study [16], 321 respondents were recruited from the visitors of inpatients using quota sampling to match the structure of age and sex in the general population. Apart from demographical questions and warm-up tasks, each respondent valued 23 hypothetical EQ-5D-3L health states using TTO during an assisted pen-and-paper interview. In the present paper, I used 6939 observations available after data cleaning procedures as described in [24].

As many as 88 respondents (29%) did not value the pits state as the worst one. The mean and median of the excessive disutility of other-than-pits state amounted to 0.3 and 0.15, respectively. In the econometric analysis, the rescaling was done using the largest disutility reported. There were 79.3% of respondents who valued the worst health state presented to them (not necessarily pits state) as strictly worse than dead, and only 1.3% exactly at 0. In 99.3% respondents, the value of the pits state (33333) was available. The average scaled disutility of dead amounts to 0.688.

The results of econometric and Bayesian approaches to estimation are presented in Table 2. In all the models, the intercept was used, as it is typically needed in EQ-5D-3L data. The results of the standard modeling differ slightly from the findings of the original valuation study [16], since in that study a slightly different approach to data cleaning was used and more observations were removed for modeling.

**Table 2:** Results for EQ-5D-3L. In subsequent parts: disutilities for dimensions/levels; utilities for 22222 and 33333; % of health states with negative utility; and relative importance of individual dimensions (level 3 in a single dimension divided by level 3 values summed across all the dimensions)

Param.	OLS	OLS*	GLM	GLM*	WLS	WLS*	Bayes	Bayes*
const	0.064	0.059	0.070	0.062	0.036	0.035	0.014	0.011
MO2	0.058	0.047	0.055	0.046	0.043	0.037	0.058	0.046
MO3	0.318	0.286	0.313	0.283	0.317	0.279	0.331	0.262
SC2	0.047	0.041	0.048	0.042	0.053	0.050	0.051	0.040
SC3	0.220	0.208	0.226	0.207	0.224	0.211	0.224	0.177
UA2	0.058	0.053	0.062	0.052	0.046	0.041	0.054	0.043
UA3	0.227	0.191	0.222	0.190	0.221	0.192	0.231	0.182
PD2	0.054	0.048	0.053	0.046	0.056	0.050	0.057	0.045
PD3	0.472	0.406	0.464	0.403	0.481	0.411	0.504	0.399
AD2	0.025	0.022	0.023	0.025	0.050	0.043	0.034	0.027
AD3	0.199	0.183	0.201	0.185	0.232	0.203	0.238	0.188
22222	0.694	0.725	0.695	0.725	0.688	0.715	0.732	0.788
33333	$-0.500$	$-0.338$	$-0.49$	$-0.332$	$-0.539$	$-0.36$	$-0.542$	$-0.219$
% $u<0$	13.2%	5.3%	13.2%	5.3%	13.2%	4.9%	13.2%	3.3%
MO	22.1%	22.4%	21.9%	22.3%	21.5%	21.5%	21.7%	21.7%
SC	15.3%	16.3%	15.8%	16.3%	15.2%	16.3%	14.7%	14.7%
UA	15.8%	15.0%	15.6%	15.0%	15.0%	14.8%	15.1%	15.1%
PD	32.9%	31.9%	32.5%	31.8%	32.6%	31.7%	33.0%	33.0%
AD	13.9%	14.4%	14.1%	14.6%	15.7%	15.7%	15.6%	15.6%

Scaled models are denoted with an asterisk. *OLS* ordinary least squares, *GLM* generalized linear model (individual error term), *WLS* weighted least squares

Similarly to the EQ-5D-5L case, for all the modeling specifications, the scaling proposed in this paper reduces the disutilities assigned to individual dimensions/levels and as a result it increases the estimated utilities of health states. The proportion of health states within the EQ-5D-3L descriptive system that are assigned negative utility decreases from approx. 13% to 3%–5%, depending on the modeling approach. These proportions should not be compared with the one for EQ-5D-5L due to the differences in the descriptive system and the structure of health states available in both systems. Similarly to the EQ-5D-5L case, there is barely any impact of the proposed correction on the relative importance of dimensions.

As a side note, we can observe that accounting for heteroscedasticity depending on state severity conveniently reduces the value of the constant (WLS) and using Bayesian estimation reduces it further.
